# Supplementary material for: regCOVID: Tracking publications of registered COVID-19 studies
Source: Res Sq. 2021 Sep 21:rs.3.rs-905657. Preprint. [Version 1] doi: 10.21203/rs.3.rs-905657/v1 (PMC8475971; doi:10.21203/rs.3.rs-905657/v1)
Supplement: Supplement 1 [file cab66fd438e4165776f4c215.docx]

# Supplemental S1

## Trials in results writing phase

We acknowledge that it takes time to write and publish results articles. As such we analyzed studies in a results writing phase based on how recently the trial has completed, based on the listed primary completion date of the trial. Supplemental Table 1 shows how many trials were completed only recently and lack result articles because they are in the results writing phase. There is no mandate to publish result articles, as only the deposition of basic summary results to the CTG registry is regulated. Supplemental Table 1 shows the number of trials when looking at various lengths of the writing period added after the trial completion date to allow for time to write the results and for journal publication (or preprint posting). Of the 1 720 interventional trials past their primary completion date, almost two-thirds (65.8%, 1 133 trials) are more than 120 days past the primary completion date, while only 257 (22.7%) of those have at least one non protocol article. 80 of 295 trials (27.1%) that are more than a year past their completion date have published result articles.

**Supplemental Table 1.** Number of trials in results writing phase.

| Days since completion | Trials with 0 result articles | Trials with at least 1 result article | Total number of trials |
| --- | --- | --- | --- |
| Less than or equal to 30 days | 9 | 1 | 10 |
| Less than or equal to 60 days | 260 | 29 | 289 |
| Less than or equal to 90 days | 392 | 50 | 442 |
| Less than or equal to 120 days | 516 | 71 | 587 |
| Less than or equal to 180 days | 710 | 116 | 826 |
| Less than or equal to 365 days | 1 177 | 248 | 1 425 |
| More than 365 days | 115 | 80 | 295 |
